# Laryngectomy in Young Patients: A Case Series and Review of the Literature

**DOI:** 10.7759/cureus.50099

**Published:** 2023-12-07

**Authors:** Leyla Ozbek, Ankit Patel, Jonathan Hughes

**Affiliations:** 1 Head and Neck Surgery, University College Hospital, London, GBR; 2 Otolaryngology, University College Hospital, London, GBR

**Keywords:** alcohol use, smoking tobacco, squamous cell carcinoma (scc), human papilloma virus dna, head and neck neoplasms, salvage total laryngectomy, total laryngectomy

## Abstract

Background and objective

The peak incidence of laryngeal cancer is seen in individuals aged over 65 years, with very few patients under 50 years developing advanced laryngeal cancer necessitating laryngectomy. Apart from often delayed diagnosis, this younger cohort faces a unique set of challenges related to fertility preservation, lower recruitment to clinical trials, and significant psychological impact. In light of this, this case series aimed to examine the various characteristics of patients below the age of 50 years undergoing total laryngectomy.

Methods

We reviewed departmental records at the University College Hospital London, spanning a period of 10 years, to identify patients who underwent total laryngectomy under the age of 50 years.

Results

The group comprised a total of nine patients over the age of 10 years: five males and four females. Six (66.7%) patients were smokers, and two (22.2%) had human papillomavirus (HPV)-16-positive disease. These patients underwent a variety of operative techniques. The length of postoperative inpatient stay varied greatly, ranging from five to 44 days (mean: 23 days).

Conclusion

There appears to be a lower prevalence of classical risk factors in our younger cohort undergoing total laryngectomy, as well as a reduced incidence of HPV-16 and a higher proportion of females. We also bring to light the significant psychological impact that these younger patients face and highlight the key learning point that clinicians must be vigilant in investigating younger patients with suspicious symptoms, even in the absence of obvious risk factors. Although further research is needed, this series is unique in that currently there are no other papers outlining laryngectomies in a patient group aged below 50 years.

## Introduction

Total laryngectomy is a surgical procedure whereby the larynx is entirely removed and the trachea is sutured to the skin, thereby forming a stoma for respiration. Total laryngectomy is primarily employed as the curative treatment for advanced laryngeal malignancy; however, the procedure also has a role in the treatment of several other conditions, including intractable laryngeal stenosis, chronic life-threatening aspiration, and recurrent respiratory papillomatosis with the risk of tracheal invasion [1]. The larynx serves many major functions, which include vocalisation, respiration, and protection of the airway via protective reflexes [2]. Squamous cell carcinomas (SCC) account for over 98% of laryngeal malignancies, with chondrosarcomas, leiomyosarcoma, and melanomas comprising a smaller number (2-5%) [3].

The two major risk factors for the development of laryngeal cancer are smoking and alcohol consumption; up to 66% of new diagnoses involve current smokers, with nearly 75% of patients having had a smoking habit in the previous 30 years [4]. Furthermore, excessive alcohol use has been associated with a six-fold increase in the development of this malignancy, with 43% of the patients reportedly consuming alcohol daily [5]. The current incidence of laryngeal malignancy is highest in Europe (5.45 new cases per 100,000) and lowest in Africa (0.68 new cases per 100,000), with a five-fold higher incidence among men versus women. The incidence and prevalence have both increased over the past 30 years, at 12.0% and 23.8% respectively, and the cumulative financial burden of laryngeal cancer is estimated to be £92.4 million in the United Kingdom. Of particular note, the incidence peaks at over 65 years in both sexes [6].

The cornerstone of the management of laryngeal cancer involves maintaining laryngeal function while ensuring adequate treatment. The treatment of laryngeal malignancy is dependent on several factors, primarily the TNM staging of the disease, but the importance of further compounding factors, such as patient comorbidities, should not be underestimated. The study by Jones et al. offers a comprehensive guideline for the management of head and neck cancer, which can be utilised by all members of the multi-disciplinary team (MDT) [7]. The postoperative course can be affected by delayed healing in patients who have had neo-adjuvant chemoradiotherapy or those with comorbidities such as diabetes mellitus [8]. Patients and their carers are tasked with navigating the complex postoperative period, which involves adjustment to the loss of speech, taste, nutrition, and humidification [9], as well as overcoming the psychological burden such a patient may understandably face. The gold standard for voice replacement is via trachea-oesophageal shunt prosthesis [10], and appropriate nutritional support is integral to the care of head and neck cancer patients [11].

Although scarce data exists, some evidence suggests that younger patients usually present in the later stages of disease in laryngeal malignancy, although when survival is stratified for early versus late disease, there is no significant difference in patient outcomes [12]. Conversely, younger patients diagnosed with breast or gastric cancer have been found to suffer from worse outcomes due to a more aggressive disease course coupled with delayed diagnosis [13]. It is important to bear in mind the medical and psychosocial challenges this younger cohort may face in comparison to their older peers; besides the frequently late diagnosis, these patients face challenges related to fertility preservation, reduced recruitment to clinical trials, and significant psychological impact [14,15].

The peak incidence of laryngeal cancer is seen in patients aged over 65 years. As such, the majority of total laryngectomies are performed in this age group. Very few patients develop advanced laryngeal cancer necessitating laryngectomy under the age of 50 years. In this case series, we aim to describe the demographic, clinical, and histopathologic features and treatment outcomes in patients under the age of 50 years undergoing total laryngectomy.

This article was previously presented as a poster at the British Academic Conference of Otorhinolaryngology, Birmingham, UK, on February 15, 2023.

## Materials and methods

We reviewed operative and departmental records at the University College Hospital, London over a 10-year period, intending to identify patients who underwent total laryngectomy under the age of 50 years. The inclusion criterion was any patient under the age of 50 undergoing total laryngectomy; these patients were identified from a database maintained by one of the authors. Patients undergoing simultaneous additional procedures, such as neck dissection, were also included. Patients with histology other than SCC were excluded. We retrospectively collected data on risk factors, comorbidities, TNM staging, human papillomavirus (HPV)-16 status, and chemoradiotherapy and surgery performed, by reviewing our electronic hospital records. We also assessed the postoperative outcomes including length of inpatient stay, postoperative complications, voice rehabilitation, nutrition, length of follow-up, recurrence rate, and survival.

## Results

We identified five males and four females ranging in age from 27 to 49 years. Six patients (66.7%) were smokers, and three patients consumed alcohol, although alcohol consumption was not documented for three patients. The patients suffered from a range of comorbidities (Table 1), with one patient developing laryngeal malignancy following the extension of a lateral tongue and oropharyngeal SCC. All patients were found to have SCC on histopathological analysis, with only two patients found to be HPV-16-positive. Eight patients had T4 disease, with varying levels of nodal involvement and no metastases at the time of intervention; the remaining patient had initially had T2 disease, which had recurred some years after initial treatment with radiotherapy. The procedures in all patients except one were performed as primary laryngectomies, with one undergoing salvage laryngectomy, and none were performed for functional purposes. Unfortunately, four patients had a recurrence of the disease following laryngectomy, all of whom are now deceased.

**Table 1:** Patient demographics, disease characteristics, and treatment details

Characteristics	Number of patients (%)
Sex	
Male	5 (55.6)
Female	4 (44.4)
Smoking	
Yes	6 (66.7)
No	3 (33.3)
Alcohol consumption	
Yes	3 (33.3)
No	3 (33.3)
Not documented	3 (33.3)
Primary tumour site	
Hypopharynx	2 (27.2)
Larynx	5 (55.6)
Supraglottis	2 (27.2)
T classification	
T2	1 (11.1)
T4	8 (88.9)
N classification	
N0	4 (44.4)
N2	4 (44.4)
N3	1 (11.1)
HPV-16 status	
Positive	2 (27.2)
Negative	7 (72.8)
Complimentary treatments	
Adjuvant chemoradiotherapy	6 (61.7)
Adjuvant radiotherapy	2 (27.2)
Neo-adjuvant radiotherapy	1 (11.1)
Type of surgery	
Primary	8 (11.1)
Salvage	1 (88.9)
Recurrence post-laryngectomy	
Yes	4 (44.4)
No	5 (55.6)
Voice rehabilitation	
Tracheo-oesophageal puncture	5 (55.6)
Electrolarynx	1 (11.1)
Oesophageal	1 (11.1)
Nil	1 (11.1)
Not documented	1 (11.1)

Six patients received adjuvant chemoradiotherapy, with one patient receiving neoadjuvant radiotherapy (RT), and two patients receiving adjuvant RT. Seven patients underwent total laryngectomy, one underwent laryngopharyngectomy, and one patient underwent pharyngolaryngo-oesophagectomy. Seven patients received bilateral neck dissections, with one receiving unilateral selective neck dissection. Gastric pull-up was performed in two patients, and primary tracheo-oesophageal puncture in three. Two patients also underwent pectoralis major flap reconstructions. Length of inpatient stay varied greatly among the patients, from five to 84 days (mean: 29 days), although the length of postoperative inpatient stay ranged from five to 44 days (mean: 23 days). Patients experienced a range of postoperative complications, including two incidences of anastomotic leak; of note, three patients did not experience any specific complications. 

One patient was followed up at a different hospital, and the details of her rehabilitation are not available. Five patients vocalised postoperatively using tracheo-oesophageal voice prosthesis, one via oesophageal voice, and one via electrolarynx, with one patient unable to vocalise at present. Six patients have been able to resume normal oral intake, with one patient receiving nutrition via a radiographically inserted gastrostomy, and one via jejunostomy. The length of follow-up has been variable, and in four patients, it has been cut short as they died in the interim period. A further four patients are still alive, with one lost to follow-up at a different hospital.

## Discussion

Laryngeal cancer in young patients is uncommon, with less than 10% of the diagnosed individuals being younger than 40 years of age [16]. Although the link between the disease and alcohol consumption and smoking is undeniable in the older patient group, the presence of classical risk factors is less prominent in younger patients [16,17], which may be partially attributable to the lower length of exposure to these aggressors, bringing to light other variables which may play a greater role in the development of laryngeal cancer in younger patients. Of note, 66.7% of our patients (all under the age of 50 years) were found to be smokers, compared with 38% under 40 and 71% over 40 in a study by Nachalon et al. involving 160 patients with laryngeal SCC [12]; when looking at our patients under the age of 40 years, the incidence of smoking fell to 50%; however, it is difficult to infer any significant meaning based on this observation in such a small patient group.

Laryngeal malignancy in non-smoking, non-drinking young patients with no other risk factors (as is seen in one of our patients) has been rarely reported in the literature, and many suggestions have been made as to the possible causes; these include environmental exposure such as passive smoke inhalation or asbestos, GORD, radiation, and diet [18]. One school of thought suggests that the presence of genetic mutations may contribute to head and neck cancer in young patients in the absence of classical risk factors; Schantz et al. have noted a greater number of bleomycin-induced chromosomal breaks in the cells of young adults with head and neck SCC [19], which may contribute to an increased risk of developing multiple malignancies [20]. However, Koch et al. reviewed the clinical and molecular patterns of three groups of patients with head and neck cancer (smokers, ex-smokers, and non-smokers), discovering that tumours in the non-smoker group contained fewer genetic mutations compared to the other two groups [21]. This suggests that there are other factors at play in the pathogenesis of head and neck cancer in a younger patient cohort. Further research is required in this field to fully understand the contributing genetic factors.

Viral infections have been implicated in the pathogenesis of laryngeal malignancy - particularly infection with HPV-16, which is found in 25% of laryngeal cancers. However, the clinical significance of such an infection is not fully understood in terms of its implications for disease prevention or treatment [22], and most of our patients (77.8%) were in fact found to be HPV-16-negative. Younger patients with laryngeal cancer have also been found to have a higher rate of HIV compared to their older counterparts [16], and malignant transformation of papillomatosis has been implicated in the development of laryngeal cancer in a younger patient cohort [23], as is seen in one of our patients who experienced malignant transformation of respiratory and vocal cord papillomatosis. 

Four (44.4%) of our patients were female, which is in sharp contrast to the gender profile of laryngeal malignancy in general, with the usual incidence manifesting a male-to-female ratio of 7:1 [18], although Nachalon et al. found the ratio to be 2.6:1 in patients under 40 years of age [12]. The incidence of oral cavity and pharyngeal malignancy is currently on the rise in Europe, and although numbers among women remain low compared to men, this can be partly attributed to an increase in alcohol and tobacco use among women, as well as an increase in HPV incidence [24]. 

Length of inpatient stay varied in our patient group, ranging from five to 84 days (mean: 29 days); however, the length of postoperative inpatient stay was reduced, ranging from five to 44 days (mean: 23 days). To our knowledge, while no data exists on the outcomes of young patients undergoing laryngectomy, there have been several studies comparing the outcomes of young versus older patients with laryngeal malignancy; interestingly, in a study of 160 patients, Nachalon et al. found that when survival was stratified for early versus late disease, there was no significant difference in patient outcomes [12]. Outcomes (including overall survival, disease-free survival, and relapse rates) in young patients with laryngeal malignancy vary across studies, with some suggesting a poorer prognosis [25], whilst others suggest an improved prognosis compared to an older patient group [26,27]. Overall, it appears that laryngeal cancer behaves in a similar pattern regardless of the patient’s age. 

The psychological impact of undergoing a total laryngectomy at a young age must not be underestimated and these patients require adjustment to the loss of speech, taste, and nutrition. These are some of the issues that compound the psychological burden that these patients face, and although a recent systematic review and meta-analysis revealed a surprising level of resilience among this patient group, a significant number suffered with the uncertainty of recurrence, disruption to daily life, and a diminished sense of self-worth [28]. Furthermore, a study by Lee-Preston et al. showed that younger patients scored worse on emotional well-being and anxiety in the 12 months following laryngectomy, compared with their older counterparts [29], highlighting the need for more intensive support. Staff should receive communication skills training, and adopt a patient-centred approach, which utilises regular psychological assessment and appropriate information-giving and support, to help ameliorate such difficulties [30]. 

This study has a few limitations; firstly, due to the rarity of this condition, our patient cohort was relatively small, which makes it difficult to infer a significant meaning from the data gathered; however, given the general scarcity of data on laryngectomies in young patients, this series succeeds in shedding light on this uncommon entity. Secondly, the data was collected retrospectively, and indeed some data is missing, as one patient was followed up at a different hospital postoperatively, and we were unable to access their records.

## Conclusions

To the best of our knowledge, there are no papers outlining laryngectomies in a patient group below 50 years in the literature, which prompted us to present this unique series that highlights the demographic, clinical, and histopathologic features and outcomes of young patients undergoing total laryngectomy. There appears to be a lower prevalence of classical risk factors among our younger cohort, apart from a reduced incidence of HPV-16 and a higher proportion of females. We also bring to light the significant psychological impact that these younger patients face, and the care that must be taken by clinicians to support such patients. This study highlights the key learning point that clinicians must be vigilant when encountering younger patients with suspicious symptoms, even in the absence of obvious risk factors.
